# Climate and Environmental Changes and Their Potential Effects on the Dynamics of Chagas Disease: Hybridization in Rhodniini (Hemiptera, Triatominae)

**DOI:** 10.3390/insects14040378

**Published:** 2023-04-12

**Authors:** Amanda Ravazi, Jader de Oliveira, Fernanda Fernandez Madeira, Giovana Menezes Nunes, Yago Visinho dos Reis, Ana Beatriz Bortolozo de Oliveira, Luísa Martins Sensato Azevedo, Cleber Galvão, Maria Tercília Vilela de Azeredo-Oliveira, João Aristeu da Rosa, Kaio Cesar Chaboli Alevi

**Affiliations:** 1 Instituto de Biociências de Botucatu, Universidade Estadual Paulista “Júlio de Mesquita Filho” (UNESP), Rua Dr. Antônio Celso Wagner Zanin, 250, Distrito de Rubião Junior, Botucatu 18618-689, SP, Brazil; 2 Laboratório de Entomologia em Saúde Pública, Faculdade de Saúde Pública, Universidade de São Paulo (USP), Av. Dr. Arnaldo 715, São Paulo 01246-904, SP, Brazil; 3 Laboratório de Biologia Celular, Instituto de Biociências, Letras e Ciências Exatas, Universidade Estadual Paulista “Júlio de Mesquita Filho” (UNESP), Rua Cristóvão Colombo 2265, São José do Rio Preto 15054-000, SP, Brazil; 4 Laboratório Nacional e Internacional de Referência em Taxonomia de Triatomíneos, Instituto Oswaldo Cruz (FIOCRUZ), Av. Brazil 4365, Pavilhão Rocha Lima, Sala 505, Rio de Janeiro 21040-360, RJ, Brazil; 5 Laboratório de Parasitologia, Faculdade de Ciências Farmacêuticas, Universidade Estadual Paulista “Júlio de Mesquita Filho” (UNESP), Rodovia Araraquara-Jaú km 1, Araraquara 14801-902, SP, Brazil

**Keywords:** Chagas disease vectors, *Rhodnius*, *Psammolestes*, experimental crosses

## Abstract

**Simple Summary:**

All 155 living species of triatomines are considered to be potential vectors of Chagas disease. Laboratory and field studies demonstrate that these insects are capable of crossing and producing hybrids. Hybrids of *Triatoma* spp. have a greater capacity and vectorial competence to acquire and transmit *Trypanosoma cruzi* than the parental species. Therefore, we conducted studies to assess whether the *Rhodnius* spp. and *Psammolestes* spp. that live in sympatry (if found in natural conditions) or allopatry (geographically isolated) are capable of producing hybrids. We observed that all cross species produce hybrid offspring in at least one direction. Although the vectorial capacity and competence of these insect vectors must be evaluated, we emphasize the importance of directing the monitoring activities of vector control programs toward possible natural hybridization events in the face of climatic and environmental changes.

**Abstract:**

Chagas disease affects about eight million people. In view of the issues related to the influence of anthropogenic changes in the dynamics of the distribution and reproductive interaction of triatomines, we performed experimental crosses between species of the Rhodniini tribe in order to evaluate interspecific reproductive interactions and hybrid production capacity. Reciprocal crossing experiments were conducted among *Rhodnius brethesi × R. pictipes*, *R. colombiensis × R. ecuadoriensis*, *R. neivai × R. prolixus*, *R. robustus × R. prolixus*, *R. montenegrensis × R. marabaensis*; *R. montenegrensis × R. robustus, R. prolixus* × *R. nasutus* and *R. neglectus* × *R. milesi*. With the exception of crosses between *R. pictipes* ♀ × *R. brethesi* ♂, *R. ecuadoriensis* ♀ × *R. colombiensis* ♂ and *R. prolixus* ♀ × *R. neivai* ♂, all experimental crosses resulted in hybrids. Our results demonstrate that both allopatric and sympatric species produce hybrids, which can generate concern for public health agencies in the face of current anthropogenic events. Thus, we demonstrate that species of the Rhodniini tribe are capable of producing hybrids under laboratory conditions. These results are of great epidemiological importance and raise an important discussion about the influence of climatic and environmental interactions on Chagas disease dynamics.

## 1. Introduction

Neglected tropical diseases (NTDs) are a diverse group of diseases prevalent in tropical and subtropical regions of 149 countries, and they affect more than one billion people living in poverty worldwide [1]. Currently, there are at least twenty pathologies considered as NTDs that can be caused by viruses (dengue, chikungunya and rabies), bacteria (Buruli ulcer, leprosy, trachoma and endemic treponematoses), parasites [Chagas disease (CD), dracunculiasis, schistosomiasis, foodborne trematodes, sleeping sickness, leishmaniasis, lymphatic filariasis, onchocerciasis, helminthiasis and taeniasis/cysticercosis], fungi (mycetoma, chromoblastomycosis and other deep mycoses), ectoparasites (scabies) and even snakebite envenomation [1].

It is estimated that NTDs kill more than 350,000 people a year [2]. In addition, they can cause disability, disfigurement, child growth impairments and impairments in human cognitive development [2,3,4,5]. Although in recent years, public health agencies have invested resources to mitigate various NTDs [6], these diseases still collectively contribute to lost productivity [7], causing harm and suffering in many countries, including several in the G20 [8].

Among the different issues that can make it difficult to control NTDs, climate and environmental changes deserve attention as they can directly influence the emergence and/or reemergence of multiple NTDs, principally those involving a vector or intermediate host for transmission (such as arboviruses, CD, schistosomiasis, sleeping sickness, leishmaniasis, lymphatic filariasis and onchocerciasis) [9,10]. Furthermore, it is considered that anthropogenic alterations can even affect – in the short (seasonal), medium (annual) and/or long (decadal) term [11]—the distribution of NTDs [10], causing changes in its transmission period and geographic expansion, since, probably, the parasites, vectors and/or hosts will be able to invade new regions and reach new human populations not yet affected by NTDs [10,11].

Climate and environmental changes can directly influence the biotic and abiotic factors involved in vector development and distribution cycles (influencing even the altitudinal and latitudinal distribution of species) [11]. Among them, temperature, for example, influences life span, reproductive aspects and feeding rates, and precipitation influences the availability of suitable habitats for reproduction [11].

Among vector-borne NTDs, CD is a disease that affects about eight million people [12,13], and it can be influenced by climate and environmental changes because factors such as increased area of dispersion of triatomines, availability of habitat, and increased mobility of vectors from the wild environment to human constructions (probably attracted to electric light [14] and encroachment into nature by humans [15,16,17,18]), which can result in high rates of infection by *Trypanosoma cruzi* (Chagas, 1909) (Kinetoplastida, Trypanosomatidae), the etiological agent of CD.

Several studies show that climate change is influencing the geographic distribution of insect vectors [19,20,21] and, consequently, the dynamics of vector-borne diseases [22,23,24]. Projections for the coming decades predict changes in the geographic distribution of triatomines and in the vectorial transmission dynamics of *T. cruzi* (Table 1) [25,26,27,28,29,30,31,32,33,34]. As a result of climate and environmental changes (such as deforestation, fires and land use), some authors consider that there may be an overlap in the distribution of triatomine species (Hemiptera, Triatominae)—the main form of transmission of *T. cruzi* [13,35]—and this may even result in the adaptation of wild species in urban and domestic environments [25,29,36].

Although the influence of man on the integrity of species, with the possibility of hybridization events between isolated species, has been known since 1930 [37], current climate and environmental changes of anthropogenic origin are generating large-scale habitat modifications (which increase the possibility of these evolutionary events occurring) [38]. Chunco [38] points out that novel hybrid zones will be able to graduate if climate change removes habitat barriers between allopatric sister taxa. However, most studies have only analyzed changes in the structure of communities influenced by climate change [39,40], with little research on the effect of climate and environmental changes on the reproductive interactions of species [38,41,42].

Currently, it is known that in addition to altering ecological processes (ranging from the decoupling of interactions between species [43] to the restructuring of entire ecosystems [44]), climate and environmental changes will have consequences for evolutionary processes as many species will face modified selection regimes with the new climates and, to avoid extinction, they must adapt or disperse to more favorable environments, which will directly influence the evolutionary processes of several species [38]. Chunco [38] points out that as these changes are reorganizing species assemblages and are breaking down physical, temporal and behavioral reproductive barriers, climate and environmental changes caused by man will probably dramatically increase the probability of hybridization for future communities, potentially resulting in introgression, speciation or even extinction events.

Currently, 158 species of triatomines are known, divided into 18 genera and five tribes [45,46,47,48], with all of them considered as potential CD vectors. The tribe Rhodniini Pinto, 1926 is a monophyletic group composed of two genera: *Rhodnius* Stål, 1859 and *Psammolestes* Bergroth, 1911 [49,50,51,52]. Members of the genus *Rhodnius* have been grouped into three major groups: *pallescens* (considered as trans-Andean group, with distribution in the west of the Andes mountain range), *pictipes* and *prolixus* (considered as cis-Andean groups, with distribution in the eastern Andes and in the Amazon) [52]. The species of the genus *Rhodnius* present a complex taxonomy; although the differentiation of the species was initially based only on morphological distinctions and similarities [53,54], the events of phenotypic plasticity and cryptic speciation make it difficult to correctly classify these vectors [55].

The analysis of the impact of climate change on the distribution of *Rhodnius* spp. demonstrates that these changes can potentially increase the range of the different species of *Rhodinus* [26,33]: Medona et al. [26] demonstrated that *R. prolixus* Stål, 1859 shows a future expansion to new areas from Venezuela, with emphasis on the current states that present low risk (Amazonas, Bolivar and Apure); in addition, models evaluated by Eberhard et al. [33] project large climatically suitable areas for *R. prolixus* in tropical regions in South America, Central America, the Caribbean, Central And East Africa, eastern Madagascar, in the south of India and Sri Lanka and throughout Southeast Asia. The projected range of *R. brethesi* Matta, 1919 and *R. ecuadoriensis* Lent & León, 1958 is limited to areas with an equatorial, tropical wet climate, namely, the Amazon region in South America and Southeast Asia (Indonesia, Malaysia, New Guinea) for *R. brethesi* and western Ecuador, parts of Indonesia, Malaysia and Papua-New Guinea as well as the Congo Basin for *R. ecuadoriensis* [33]. Furthermore, only a few regions outside the Americas with a distinctly tropical climate have been projected as climatically suitable for both species, including the Congo Basin in Central Africa and a few parts of Southeast Asia [33].

Knowledge of the reproductive interaction of members of the Rhodniini tribe is restricted to only four crosses, namely, between *R. prolixus* and *R. neglectus* Lent, 1954, *R. robustus* Larrousse, 1927 and *R. pictipes* Stål, 1872, *R. pallescens* Barber, 1932 and *R. colombiensis* Mejia, Galvão & Jurberg, 1999 and *P. tertius* Lent & Jurberg, 1965 and *P. coreodes* Bergroth, 1911 [56,57,58,59]. These crosses indicate that, possibly, there are no interspecific pre-zygotic reproductive barriers for the species of this tribe since hybrids were obtained for at least one of the directions of all crosses [56,57,58,59].

In view of the issues related to the influence of anthropogenic changes (climate and environmental changes) in the dynamics of distribution and reproductive interaction of species and considering that the knowledge about the CD vectors of the Rhodniini tribe is still incipient, we performed experimental crosses between species of this tribe in order to evaluate interspecific reproductive interactions and hybrid production capacity.

## 2. Materials and Methods

### 2.1. Experimental Crosses

In order to evaluate the reproductive compatibility [60] between the species of the genus *Rhodnius*, reciprocal crossing experiments were conducted among *R. brethesi* × *R. pictipes*, *R. colombiensis* × *R. ecuadoriensis*, *R. neivai* Lent, 1953 × *R. prolixus*, *R. robustus × R. prolixus*, *R. montenegrensis* Rosa et al., 2012 *× R. marabaensis* Souza et al., 2016, *R. montenegrensis × R. robustus, R. prolixus* × *R. nasutus* Stål, 1859 and *R. neglectus* × *R. milesi* Carcavallo, Rocha, Galvão & Jurberg, 2001. The experiments were carried out in the Triatominae Insectarium of FCFAR/UNESP, Araraquara, São Paulo, Brazil. Parental insects were randomly selected from live colonies maintained at the FCFAR/UNESP insectarium. The insects were sexed as 5th instar nymphs [61], and male and female nymphs were kept in separate pots until they performed the imaginal molt and reached the adult stage, thus ensuring that the triatomines used in the experiments were adult virgins [62]. For the crosses, five couples from each set were placed in separated plastic jars (5 cm in diameter × 10 cm in height) and kept at room temperature (average of 24 °C [63]) and an average relative humidity of 63% [63]. The crosses were maintained for 4 months. Weekly, the insects were fed with duck blood and the eggs were collected. At the end of the experiment, the egg-hatching rate was calculated.

### 2.2. Distribution Maps

The distribution maps for *Rhodnius* and *Psammolestes* species were made with QGIS 3.24 [64]. Species occurrence data were obtained from the Global Biodiversity Information Facility (GBIF) (Table 2). All available coordinates for each taxon have been included.

## 3. Results and Discussion

**Figure 1:**
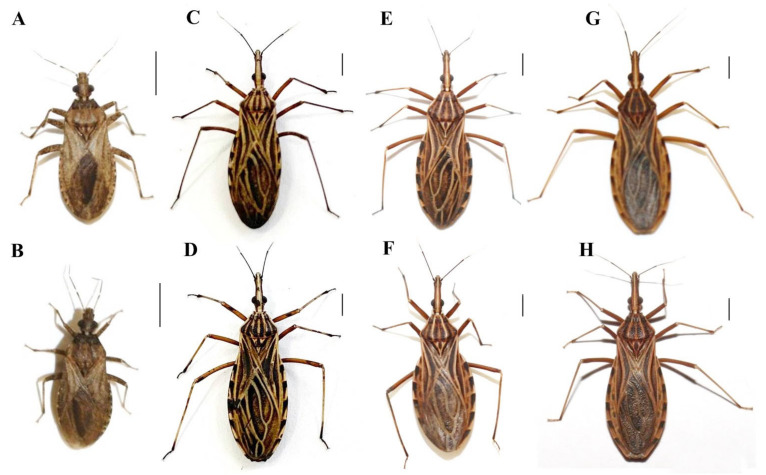
Hybrids resulting from crosses between species of the Rhodniini tribe. Hybrids resulting from crosses between *P. coreodes* ♀ × *P. tertius* ♂ (**A**), *P. tertius* ♀ × *P. coreodes* ♂ (**B**), *R. marabaensis* ♀ × *R. montenegrensis* ♂ (**C**), *R. montenegrensis* ♀ × *R. marabaensis* ♂ (**D**), *R. montenegrensis* ♀ × *robustus* ♂ (**E**), *R. robustus* ♀ × *R. montenegrensis* ♂ (**F**), *R. prolixus* ♀ × *R. nasutus* ♂ (**G**), and *R. nasutus* ♀ × *R. prolixus* ♂ (**H**). Bar: 0.5 cm.

**Figure 2:**
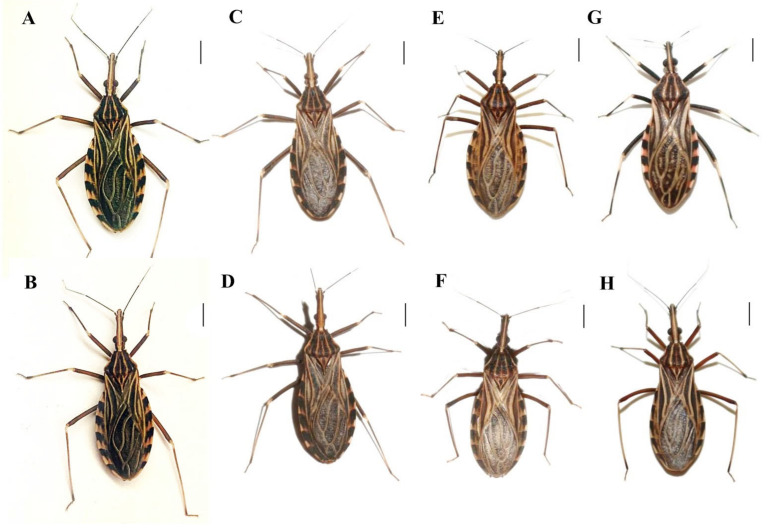
Hybrids resulting from crosses between species of the Rhodniini tribe. Hybrids resulting from crosses between *R. milesi* ♀ × *R. neglectus* ♂ (**A**), *R. neglectus* ♀ × *R. milesi* ♂ (**B**), *R. taquarussuensis* (syn *R. neglectus*) ♀ × *R. neglectus* ♂ (**C**), *R. neglects* ♀ × *R. taquarussuensis* (syn *R. neglectus*) ♂ (**D**), *R. robustus* ♀ × *R. prolixus* ♂ (**E**), *R. prolixus* ♀ × *R. robustus* ♂ (**F**), *R. brethesi* ♀ × *R. pictipes* ♂ (**G**), and *R. neivai* ♀ × *R. prolixus* ♂ (**H**). Bar: 0.5 cm.

**Figure 3:**
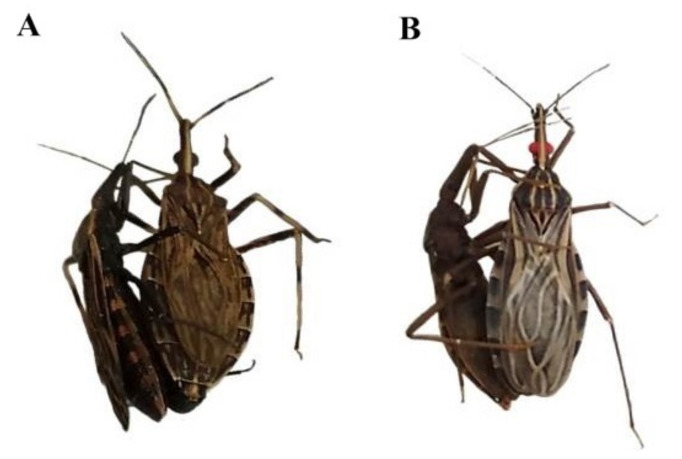
Interspecific crosses between *R. pictipes* ♀ × *R. brethesi* ♂ (**A**) and *R. prolixus* ♀ × *R. neivai* ♂ (**B**). Note: The background was removed with Adobe Photoshop CS6.

All experimental crosses resulted in hybrids (in at least one direction) (Table 3, Figure 1 and Figure 2). These data, together with data from the literature [56,57,58,59], confirm that pre-zygotic reproductive barriers are absent among most species of the Rhodniini tribe since only the experimental crosses between *R. pictipes* ♀ × *R. brethesi* ♂, *R. ecuadoriensis* ♀ × *R. colombiensis* ♂ and *R. prolixus* ♀ × *R. neivai* ♂ [even though interspecific copulations have occurred and have been observed (Figure 3)] did not result in a hybrid.

Some studies in the literature have evaluated how the current scenario associated with climate change can affect the geographic distribution of triatomine species [25,26,27,28,29,30,31,32,33,34]. However, until now, the influence of these changes on the reproductive dynamics of vectors of the Rhodniini tribe has never been discussed. Evaluating the hybridization capacity between vector species also has epidemiological implications [65,66,67,68,69,70,71] since it is known that under laboratory conditions, Triatominae hybrids may present greater vector competence than the parents [66,67,68,69,71].

Triatomines are potential vectors from the hatching of eggs, that is, all nymph stages (first- to fifth-instar nymphs) (males and females), as well as adults (males and females) are hematophagous and can transmit *T. cruzi* through faeces/urine if infected [35]. The genera *Triatoma* Laporte, 1832, *Panstrongylus* Berg, 1879 and *Rhodnius* are the most important for the epidemiology of CD [72]. *Rhodnius prolixus* is the main domestic vector of *T. cruzi* in Venezuela, Colombia, and parts of Central America [73,74], *R. pallescens* is an important vector of *T. cruzi* in Panama [75] and *R. ecuadoriensis* is an important vector in Ecuador [76,77]. In addition, other species are highlighted as vectors of secondary importance, such as *R. neglectus* [78] and *R. nasutus* [79].

Several studies carried out with *Triatoma* spp. have shown that triatomine hybrids can play an important role in the transmission of CD: Martinez-Ibarra et al. [66,67] evaluated three behaviors of epidemiological importance (the time interval for the onset of feeding, feeding time and defecation time) for five *Triatoma* species from Mexico and their hybrids and demonstrated that hybrids have more potential to acquire infection and transmit *T. cruzi* than the parental species; in addition, Martinez-Ibarra et al. [68] and Meraz-Medina et al. [69] evaluated biological parameters related to hatching, lifetime, number of blood meals to molt, percentage of females at the end of the cycle, number of laid eggs, and mortality for each instar of five *Triatoma* species from Mexico and their hybrids and observed that in four of the six studied parameters (accumulative mortality, the percentage of females, mean number of laid eggs and eggs hatching), the hybrid cohorts had better fitness results than the parental cohorts. López et al. [80] analyzed the dynamics of feeding–defecation behavior in fifth-stage nymphs and adults of hybrids resulting from the cross between *T. platensis* (Neiva, 1913) and *T. infestans* (Klug, 1834) and compared them with the parents. The authors noted that adults and fifth-instar nymphs of hybrids have a feeding and defecation behavior similar to *T. infestans*: they achieve feeding in a short time and first defecation occurs during or just after feeding. Nevertheless, the hybrid’s ingestion of blood occurs at a higher velocity and they require higher blood intake to provoke early defecations. Lastly, Vicente et al. [81] analyzed the feeding and defecation pattern of *T. rosai* Alevi et al., 2020, *T. sordida* (Stål, 1859) and the experimental hybrids and observed that there was no significant difference between the times of feeding and defecation and both parents and hybrids defecated during the blood meal.

Based on the scientific information related to the epidemiological importance of Triatominae hybrids [65,66,67,68,69,70,71,80], the authors raise some questions: i. the hybrid cohorts were more effective vectors of *T. cruzi* than their parental species, which could indicate a potentially higher risk of transmission of *T. cruzi* to reservoir hosts; ii. the increase in hybrid fitness could lead to an increase in the epidemiologic risks caused by the transmission of *T. cruzi* to humans; and iii. based on blood ingestion velocity, the amount of blood ingested, and the short time required for the production of the first defecation, the hybrids can be a competent *T. cruzi* vector.

**Figure 4:**
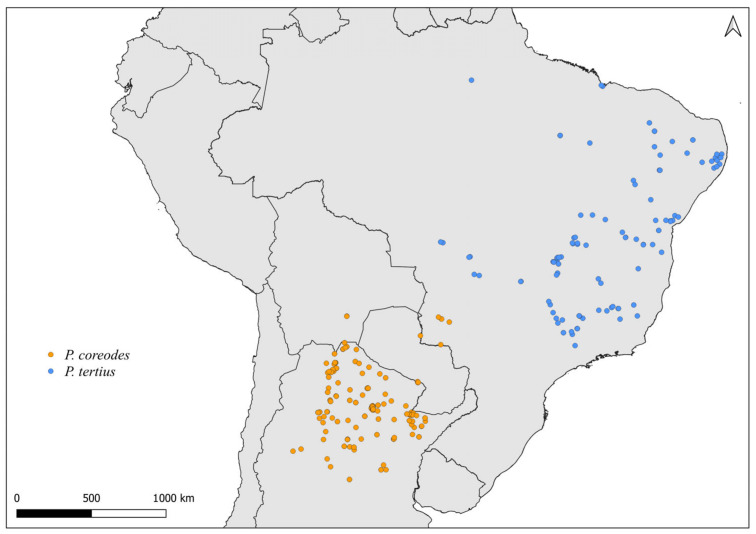
Distribution maps for *P. coreodes* and *P. tertius*.

**Figure 5:**
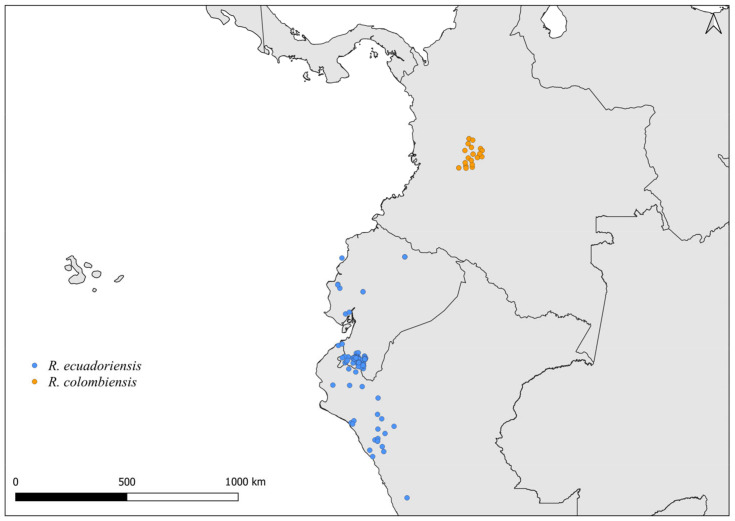
Distribution maps for *R. ecuadoriensis* and *R. colombiensis*.

**Figure 6:**
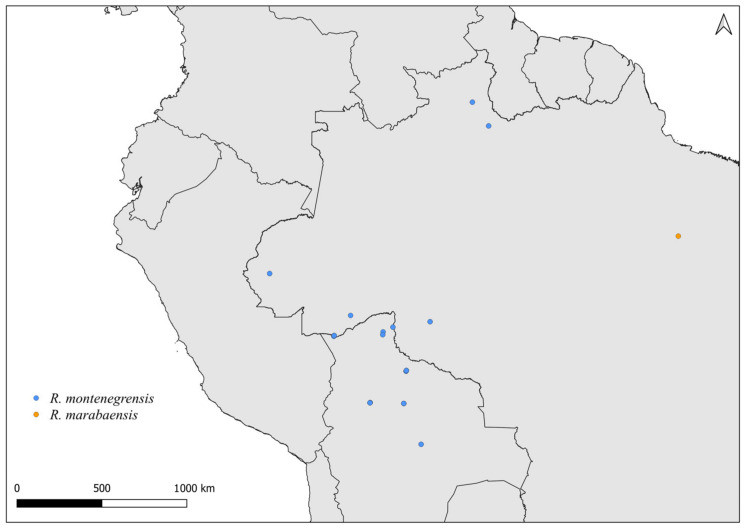
Distribution maps for *R. montenegrensis* and *R. marabaensis*.

**Figure 7:**
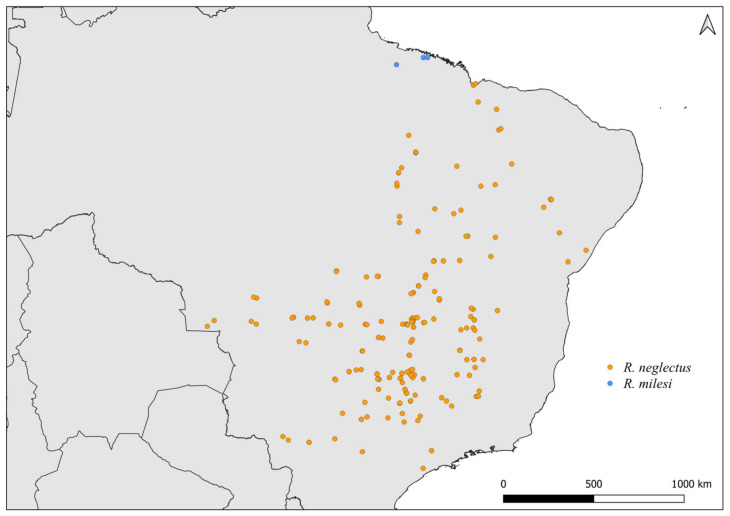
Distribution maps for *R. neglectus* and *R. milesi*.

**Figure 8:**
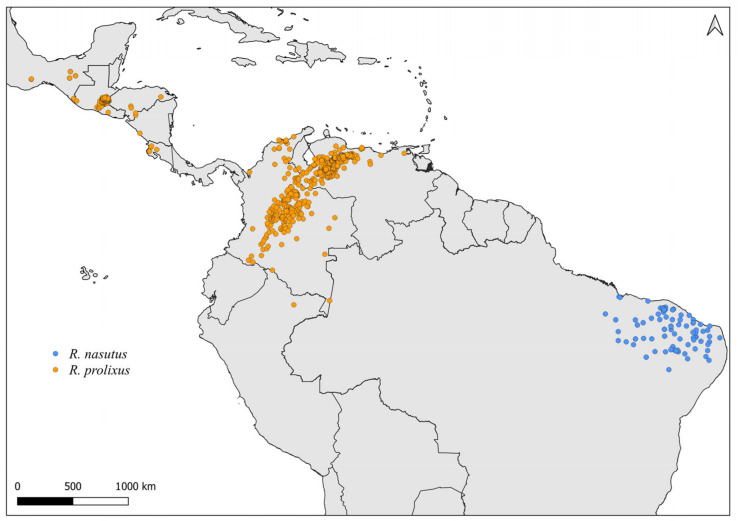
Distribution maps for *R. nasutus* and *R. prolixus*.

**Figure 9:**
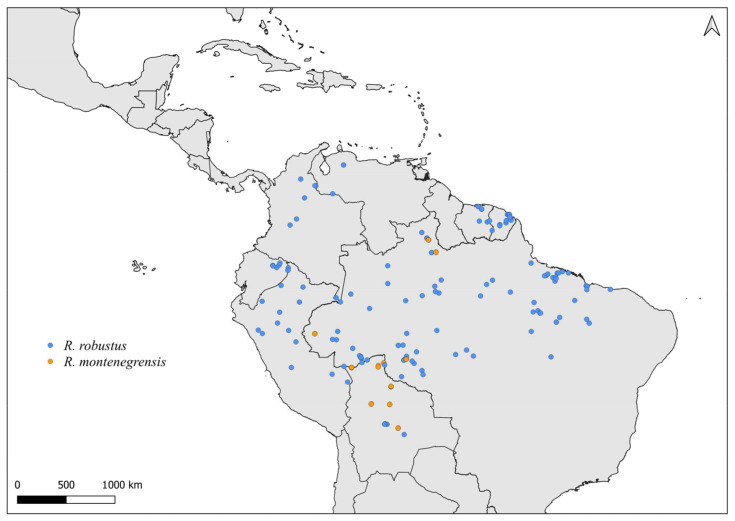
Distribution maps for *R. robustus* and *R. montenegrensis*.

**Figure 10:**
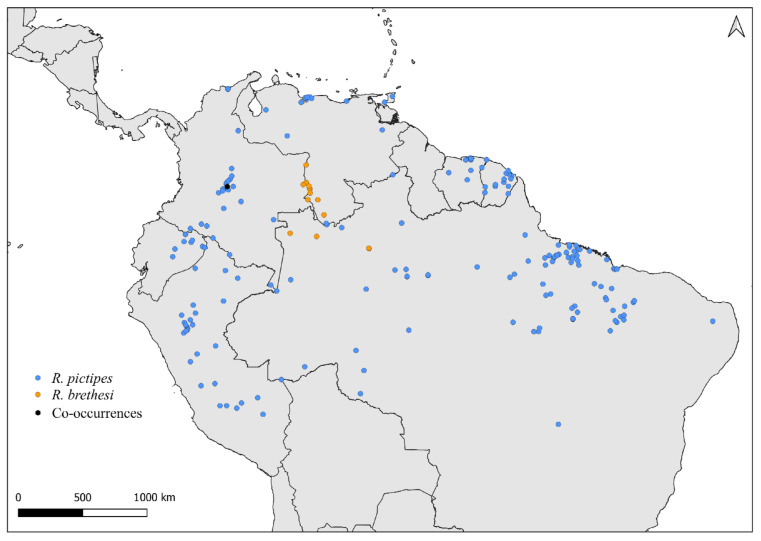
Distribution maps for *R. brethesi* and *R. pictipes*.

**Figure 11:**
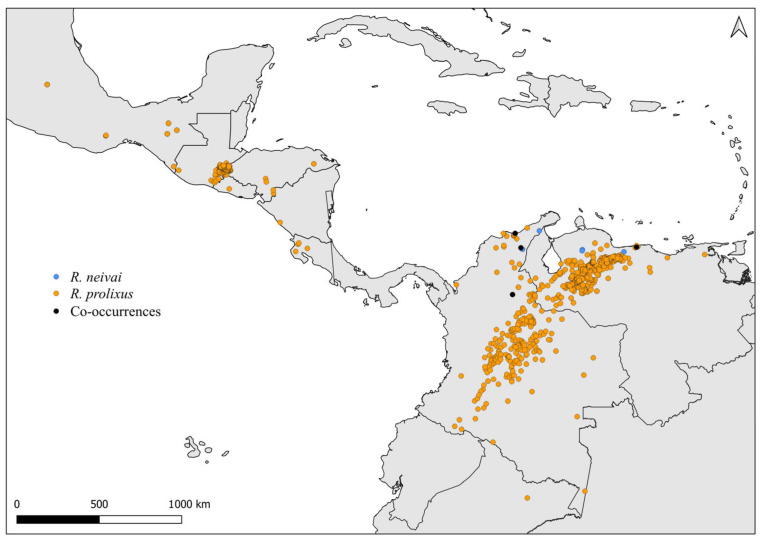
Distribution maps for *R. neivai* and *R. prolixus*.

**Figure 12:**
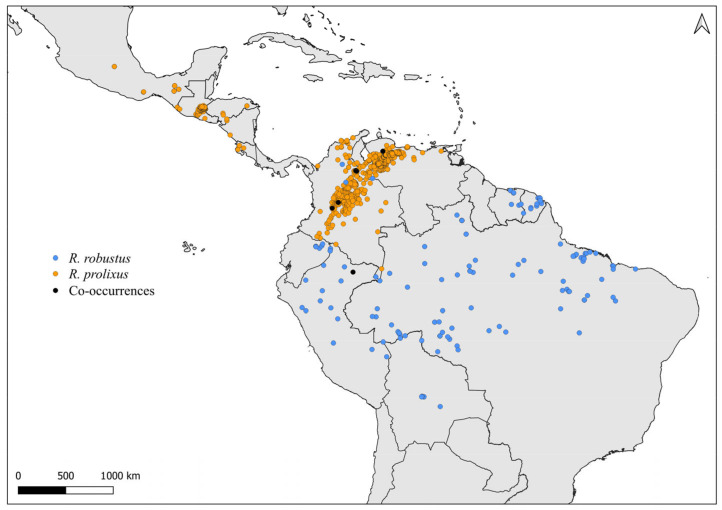
Distribution maps for *R. prolixus* and *R. robustus*.

Faced with climate and environmental changes, reorganizations in the distribution of species are taking place, which can increase the probability of encounters between allopatric species under natural conditions [38]. Ecological niche modeling studies, which make it possible to predict the effects of climate variations on biodiversity (and, in the case of CD vectors, it allows the prediction of areas at risk of transmission of *T. cruzi*), demonstrate that the potential geographic distribution of triatomines may also result in encounters between allopatric species in possible new distribution areas [31,82]. Our results demonstrate that both allopatric (Figure 4, Figure 5, Figure 6, Figure 7, Figure 8 and Figure 9) and sympatric (Figure 10, Figure 11 and Figure 12) *Rhodnius* species produce hybrids (Table 3), which can generate concern for public health agencies in the face of current anthropogenic events.

Introgression (or “introgressive hybridization”) describes the incorporation (usually via hybridization and backcrossing) of alleles from one entity (species) into the gene pool of a second, divergent entity (species) [83,84]. Among the possible events associated with the phenomenon of hybridization in vector insects, introgression has evolutionary and, above all, epidemiological importance [85,86]. This phenomenon can allow genes related to the capacity and/or vector competence of the main vector species of CD to be transferred to wild species (thus increasing the number of vectors of primary importance).

The possibility of hybrid formation between *Rhodnius* species, demonstrated under experimental conditions (Table 3), highlights the need for attention from the scientific community and, mainly, of health agents since the *Rhodnius* taxonomy, which is already complex due to phenotypic plasticity events and cryptic speciation [55], could become even more complex if natural hybrids begin to be collected by residents or in entomoepidemiological surveys.

Souza et al. [87] highlight that important changes in the epidemiology of CD may occur as a result of environmental changes produced by deforestation, changes in land cover and unplanned urbanization and emphasize that they do not expect changes in the epidemiology of CD as a direct consequence of climate change in the short term. Our results, on the other hand, demonstrate that the hybridization capacity of the species of the Rhodniini tribe can be a worrying factor in the short term in the face of climate change; hence, it is necessary to intensify the control systems and continuous surveillance of triatomines.

## 4. Conclusions

Here, we demonstrate that species of the Rhodniini tribe can produce hybrids under laboratory conditions. These results are of great epidemiological importance and raise an important discussion about the influence of climatic and environmental interactions on CD dynamics, emphasizing the need to carry out studies to assess the capacity and vectorial competence of hybrids of *Rhodnius* spp.

## Figures and Tables

**Table 1:** Potential impact of climate change on the geographical distribution of triatomines and in the vectorial transmission dynamics of *T. cruzi*.

Species	Study Area	Projection Periods	Key Findings	Reference
*Triatoma sanguisuga*, *T. barberi*, *T. peninsularis*, *T. protracta*, *T. rubida*, *T. brailovskyi*, *T. gerstaeckeri*, *T. longipennis*, *T. mazzottii*, *T. mexicana*, *T. pallidipennis*, *T. phyllosoma*, *T. picturata*, *T. recurva*, *T. dimidiata*, *Eratyrus cuspidatus*, *Paratriatoma hirsuta* and *P. lecticularia*	North America	2050 2070	1. Significant alterations in risk of vector-borne *T. cruzi* transmission; 2. Impacts of climate change on *T. cruzi* vector species and their geographical locations; 3. Need for effective vector surveillance to aid in CD prevention and control.	[28]
*T. gerstaeckeri* and *T. sanguisuga*	North America	2050	1. A potential change in the distribution of both species is predicted due to climate change, indicating an increase in *T. cruzi* transmission risk by the year 2050; 2. Predicted shift of CD risk due to climate change.	[29]
*P. lecticularia, T. protracta* and *T. sanguisuga*	United States	2030	1. The increase in temperature is expected to promote the expansion of the vector range by the year 2030; 2. Potential for CD to emerge in the country; 3. It is believed that interdisciplinary collaboration is necessary to increase the accuracy of future disease risk predictions.	[30]
*Panstrongylus geniculatus, E. mucronatus, Rhodnius prolixus, R. robustus* and *T. maculata*	Venezuela	2020 2060 2080	1. Global climate change is predicted to slightly decrease the overall vulnerability of the population to *T. cruzi* vector species; 2. It is believed that fewer citizens will be exposed to *T. cruzi* vectors over the next 50–70 years; 3. Predictions can enhance the ability to prevent and control CD transmission in Venezuela in the future.	[25]
*R. prolixus* and *T. infestans*	VenezuelaArgentina	2050	1. Forecasts reveal the increased expansion of *R. prolixus* to new areas and a decrease in the current geographical range of *T. infestans*; 2. Overall study conclusions reflect a lower incidence of CD infections by 2050.	[26]
*T. brasiliensis brasiliensis*, *T. b. macromelasoma*, *T. juazeirensis*, *T. sherlocki* and *T. melanica*	Brazil	2020 2050	1. Projection of little change in species’ distributions under future climate change scenarios; 2. *T. b. brasiliensis* has the greatest distributional potential to colonize new areas; 3. Conclusions may help guide proactive surveillance and control activities to reduce the risk of future CD transmission.	[31]
*T. infestans*	Chile	2070	1. The distribution under two global climate change scenarios showed low variation, with a minimal reduction tendency in suitable areas; 2. Climate change appears to play a major role in the reemergence of CD and of the vector in the country; 3. The impact of temperature and precipitation on the distribution of *T. infestans* indicates the need for aggressive vector control efforts in Chile.	[32]
*M.epraia spinolai* and *M. gajardoi*	Chile	2070	1. Under future climate conditions, these species could modify their potential geographical range; 2. The suitable areas for both species may be greater than currently known, generating new challenges in terms of vector control and prevention; 3. Preventive measures to avoid accidental human vectorial transmission by wild vectors of *T. cruzi* become critical, considering the uncertainty of the future suitable areas projected in this study.	[27]
*P. geniculatus, P. megistus, R. brethesi, R. ecuadoriensis, R. prolixus, T. b. brasiliensis, T. dimidiata, T. infestans, T. maculata, T. rubrofasciata* and *T. sordida*	Different countries from five continents: Africa, Asia, Europe, Oceania and America.	2030	1. Global species distribution modeling revealed several regions with current suitable climatic conditions; 2. *R. brethesi, R. ecuadoriensis* and *T. maculata* are limited to one or a few areas with mostly tropical climate; 3. *T. b. brasiliensis, P. geniculatus, P. megistus, R. prolixus, T. dimidiata* and *T. rubrofasciata* find suitable climate conditions in a broad range of tropical and subtropical regions; 4. *T. sordida* and *T. infestans* possess a broad potential range in temperate regions; 5. It may be beneficial to establish national and international vector surveillance programs to monitor the spread of vectors and to register CD as a reportable disease.	[33]

**Table 2:** List of occurrence datasets for each species obtained from GBIF.

Species	GBIF Dataset
*Psammolestes coreodes* Bergroth, 1911	https://doi.org/10.15468/dl.vhbk9r
*Psammolestes tertius* Lent & Jurberg, 1966	https://doi.org/10.15468/dl.d78jfs
*Rhodnius brethesi* Matta, 1919	https://doi.org/10.15468/dl.hctjyp
*Rhodnius colombiensis* Mejia, Galvão & Jurberg, 1999	https://doi.org/10.15468/dl.muvwy2
*Rhodnius ecuadoriensis* Lent & León, 1958	https://doi.org/10.15468/dl.bbc7ft
*Rhodnius marabaensis* Souza et al., 2016	https://doi.org/10.15468/dl.v32gdm
*Rhodnius milesi* Carcavallo, Rocha, Galvão & Jurberg, 2001	https://doi.org/10.15468/dl.ga2kks
*Rhodnius montenegrensis* da Rosa et al., 2012	https://doi.org/10.15468/dl.7gjaws
*Rhodnius nasutus* Stål, 1859	https://doi.org/10.15468/dl.e8vm73
*Rhodnius neglectus* Lent, 1954	https://doi.org/10.15468/dl.9w7xdu
*Rhodnius neivai* Lent, 1953	https://doi.org/10.15468/dl.zghcuf
*Rhodnius pictipes* Stål, 1872	https://doi.org/10.15468/dl.tknw8g
*Rhodnius prolixus* Stål, 1859	https://doi.org/10.15468/dl.ggavgu
*Rhodnius robustus* Larrousse, 1927	https://doi.org/10.15468/dl.bcps8k

**Table 3:** Interspecific crosses performed in the Rhodniini tribe. * Data taken from Ravazi et al. [59].

Rhodniini	Crossing Experiments	Eggs	Hybrids	Hatch Rate
*Psammolestes*	*P. coreodes* ♀ × *P. tertius* ♂	54 *	23 *	43% *
	*P. tertius ♀ × P. coreodes ♂*	117 *	30 *	26% *
*Rhodnius*	*R. marabaensis ♀ × R. montenegrensis ♂*	214	128	60%
	*R. montenegrensis ♀ × R. marabaensis ♂*	436	36	08%
	*R. brethesi ♀ × R. pictipes ♂*	268	16	06%
	*R. pictipes ♀ × R. brethesi ♂*	162	00	00%
	*R. colombiensis ♀ × R. ecuadoriensis ♂*	170	45	06%
	*R. ecuadoriensis ♀ × R. colombiensis ♂*	42	00	00%
	*R. montenegrensis ♀ × robustus ♂*	565	331	59%
	*R. robustus ♀ × R. montenegrensis ♂*	611	484	57%
	*R. neivai ♀ × R. prolixus ♂*	70	22	31%
	*R. prolixus ♀ × R. neivai ♂*	368	00	00%
	*R. prolixus ♀ × R. nasutus ♂*	318	71	22%
	*R. nasutus ♀ × R. prolixus ♂*	316	14	04%
	*R. milesi ♀ × R. neglectus ♂*	357	81	23%
	*R. neglectus ♀ × R. milesi ♂*	366	314	86%
	*R. robustus ♀ × R. prolixus ♂*	622	447	72%
	*R. prolixus ♀ × R. robustus ♂*	437	221	51%
	*R. taquarussuensis* (syn. *R. neglectus*) *♀ × R. neglectus ♂*	540	496	92%
	*R. neglects ♀ × R. taquarussuensis* (syn. *R. neglectus*) *♂*	737	636	85%

## Data Availability

All relevant data are within the manuscript.
